# Retrospective Investigation of a Lead Poisoning Outbreak from the Consumption of an Ayurvedic Medicine: Durban, South Africa

**DOI:** 10.3390/ijerph120707804

**Published:** 2015-07-10

**Authors:** Angela Mathee, Nisha Naicker, June Teare

**Affiliations:** 1 Environment & Health Research Unit, Medical Research Council, P.O. Box 87373, Houghton 2041, Johannesburg, Gauteng, South Africa; E-Mails: nisha.naicker@mrc.ac.za (N.N.); june.teare@mrc.ac.za (J.T.); 2 Faculty of Health Sciences, University of Johannesburg, P.O. Box 17011, Doornfontein 2018, Gauteng, South Africa; 3 School of Public Health, University of the Witwatersrand, 27 St Andrews Road, Parktown 2193, Johannesburg, South Africa

**Keywords:** lead poisoning, Ayurvedic, traditional medicine, South Africa

## Abstract

Ayurvedic medicines have been gaining in popularity around the world in recent decades, but have also been associated with lead contamination and poisoning. In 2012 in Durban, South Africa, a lead poisoning outbreak among adolescents was associated with the consumption of an Ayurvedic medicine for the treatment of skin conditions. In 2014 eight individuals (out of 12 affected) were traced and interviewed. Questionnaires were administered; blood samples were taken for lead content analysis; and medical records were reviewed. Samples of the implicated medicines were analyzed to determine lead levels. Blood lead levels during the acute phase ranged from 34 to 116 µg/dL; and during the current study (two years later) from 13 to 34 µg/dL. The implicated lead capsules had a lead content of 125,235 µg/g. Participants suffered a wide range of non-specific ill health symptoms; and there was a significant delay in the diagnosis of lead poisoning. This study highlights the potential for lead poisoning outbreaks from the consumption of Ayurvedic medicines in African settings. There were weaknesses with regard to the diagnosis of and response to the outbreak, for which measures need to be put in place to ensure greater awareness of the role of Ayurvedic medicine in lead poisoning, and prompt diagnosis and treatment of future cases.

## 1. Introduction

Ayurvedic medicine has been practiced in India for millennia, but in recent decades, facilitated in part by Internet-based marketing and sales, there has been a global upsurge in its use [1]. Many Ayurvedic medicines are comprised of herbs, but some production processes, such as *rasa shastra*, may incorporate the use of minerals and metals [2] or animal products [3]. A study undertaken in the United States of America showed that around one fifth of Ayurvedic medicines available in the Boston area had levels of lead, mercury, and/or arsenic that exceeded national guideline levels. The median lead content in 13 contaminated samples of Ayurvedic medicine was 40 µg/g, ranging from 5 to 37,000 µg/g [4]. Ayurvedic medicines have been implicated in numerous cases of lead poisoning [5,6,7], including fatal cases of encephalopathy, status epilepticus, congenital paralysis, and developmental delay [4]. Despite growing recognition of the role of Ayurvedic medicines in lead or other forms of metal poisoning, there are limited regulations or control mechanisms in place in this regard in many parts of the world, and there have been calls for further studies to characterize the extent of the problem [8].

In 2012, 12 people in Durban, South Africa were involved in a lead poisoning outbreak, with blood lead levels ranging up to 116 µg/g. The source was determined to be an Ayurvedic treatment for skin problems. Two years after the outbreak, a study was undertaken amongst eight traceable subjects to retrospectively gain an understanding of the factors involved in, and public health response to, the outbreak, and the current blood lead concentrations and health status of those affected. This, to our knowledge, is the first published account of an outbreak of lead poisoning in an African setting associated with Ayurvedic medicines. Consideration of the factors and key agency responses to the outbreak may inform policy and approaches to a public health problem of growing importance in South Africa, as well as on a global level.

## 2. Methods

Through a networking process eight subjects and/or their parents were traced, and appointments for interviews scheduled. Four additional people known to have consumed the Ayurvedic treatment had emigrated, and did not participate in the current study. Pre-structured questionnaires were administered and free-ranging interviews conducted with study participants, their parents/families, and relevant stakeholders (such as local environmental health practitioners) to gain an understanding of the unfolding of the incident. Medical records were reviewed to obtain the results of blood lead tests and chelation treatments administered. Blood lead and hemoglobin tests were offered to subjects and family members. Samples of the implicated Ayurvedic medicine were dispatched to an analytical laboratory at the National Institute for Occupational Health in Johannesburg for lead content analysis. Metal concentrations in the herbal medicines were determined using ICPMS: Agilent ICPMS 7700 Series, fitted with Mass Hunter Software. Calibration was undertaken against two internal (in-house prepared) QCs and two CRMs TM-15.2 & TMDA-51.4 purchased from Industrial Analytical (Midrand, South Africa). For blood lead determinations an accredited method (NIOH0034) was used with the aid of a Perkin Elmer, GFAAS, Model Analyst 600. Calibration was done against two CRMs Seronorm in whole blood (L1 & L2) obtained from Industrial Analytical (Midrand, South Africa). In addition, three in-house prepared QCs were used. Approval for the study was obtained from the Ethics Committee of the South African Medical Research Council (EC002-3/2013).

## 3. Results and Discussion

### 3.1. Profile of the Study Population

Three of the eight study participants were male. At the time of the incident in August 2012, the participants were aged from 14 to 25 years, with the mean and median ages respectively equaling 16 and 14.5 years (see Table 1). All were white, spoke English as their first language, and were of relatively high socioeconomic status. Most lived in the suburb of Hillcrest, which is located on the outskirts of the city of Durban in South Africa’s KwaZulu Natal province.

### 3.2. Source and Distribution of the Product

Interviews with the participants and their parents revealed that all study subjects had used Ayurvedic medicine for the treatment of acne and skin problems. The product had been obtained from health shops, by direct marketing, or prescribed by a local homeopathic practitioner. All necessary importation procedures in South Africa had been followed. The capsules were packaged in white plastic containers, each containing 90 capsules, with the recommended maximum dose being four capsules per day. Participants had been ingesting the capsules for up to 18 months when a new batch was imported from India. Around three to four weeks after commencing use of a product from the second batch, subjects started experiencing a range of non-specific symptoms, such as headaches, abdominal pain, malaise, and fatigue, which worsened over time (see Table 1).

### 3.3. Diagnosis

There was a protracted delay in the diagnosis of lead poisoning. Most parents had observed and become concerned about the variety, and worsening, of symptoms in their ailing children around three to four weeks after commencing ingestion of the contaminated batch of Ayurvedic medicines. Despite this, over a period of six months and consulting general practitioners and a variety of medical specialists, including neurologists, orthopedic surgeons, pediatricians, hematologists, psychologists, cardiologists, endocrinologists, and gastroenterologists, and visiting emergency departments when symptoms were particularly severe, lead poisoning was never considered and no instruction for a diagnostic blood lead test was ever given in respect of any of the study subjects, even after being informed about the ingestion of an Ayurvedic treatment. Instead it was the fortuitous observation by a laboratory pathologist of basophilic stippling on a blood smear from one of the subjects that first raised the prospect of lead poisoning as an explanation for the range of generalized symptoms exhibited. Re-examination of a blood sample from a second subject, and the similar observation of blood stippling, strengthened the suspicion of a lead poisoning outbreak, leading to calls for blood lead tests in all subjects. By this time, the participants had been advised to have, or endured, a wide range of unnecessary, often invasive, diagnostic examinations, when a simple blood lead test would have sufficed. These included blood sampling, gastroscopy, colonoscopy, CT scanning, MRIs, thyroid functioning and other endocrinology testing, X-Rays, and ultrasound examinations. Prior to the conclusive diagnosis of lead poisoning, a range of diagnoses and explanations for the symptoms were offered by doctors, including depression, “burnout,” menstrual cramps, influenza, and “growing pains.”

Following the diagnosis of lead poisoning and realization of the involvement of multiple individuals, local environmental health practitioners conducted an investigation that identified an Ayurvedic medication for skin problems as a common potential source of lead. Laboratory analyses confirmed highly elevated lead concentrations. All purchasers of the product were contacted by the distributors and/or participants/their parents, resulting in all of the containers from the second batch being accounted for, and blood lead testing in all the affected individuals.

### 3.4. Blood Lead Distributions

Peak blood lead levels measured during the acute phase of the outbreak (August/September 2012) ranged from 34 to116 µg/dL, with the mean and median levels respectively equaling 73 and 74 µg/dL. South Africa currently has no blood lead surveillance program in place, and there is hence little information available to paint a picture of the national blood lead distribution in the country. What is known about lead exposure in South Africa comes from a relatively small number of local surveys undertaken in specific groups. For example, studies of first grade schoolchildren in relatively poor schools in Cape Town and Johannesburg indicated mean blood lead levels of 7 and 9 µg/dL respectively [9]. Compared to these findings, the blood lead levels of the current study subjects are exceedingly high. They are also highly elevated relative to the current reference level (5.0 µg/dL) of the US Centers for Disease Control, which they initially exceeded by a factor of 23.

**Figure 1:**
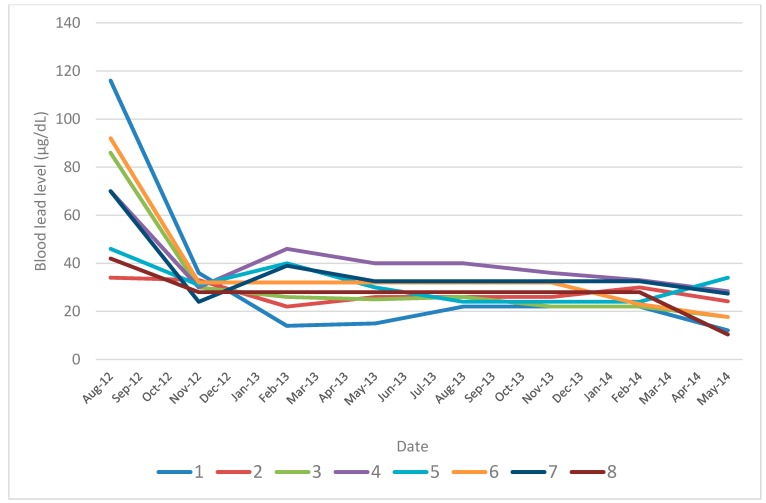
Blood lead trend by subject: 2012 to 2014.

Between August 2012 and the current study (undertaken in May 2014), participants’ blood lead concentrations declined substantially, though they remain within a range of concern (see Figure 1). Venous blood lead samples collected from the study participants during May 2014 showed that blood lead levels ranged from 13 to 34 µg/dL, with the mean and median levels respectively equaling 22 and 24 µg/dL. A more detailed breakdown of the blood lead levels is given in Table 1. The highest blood lead level is now around seven times higher than the current CDC reference level of 5 µg/dL.

**Figure 2:**
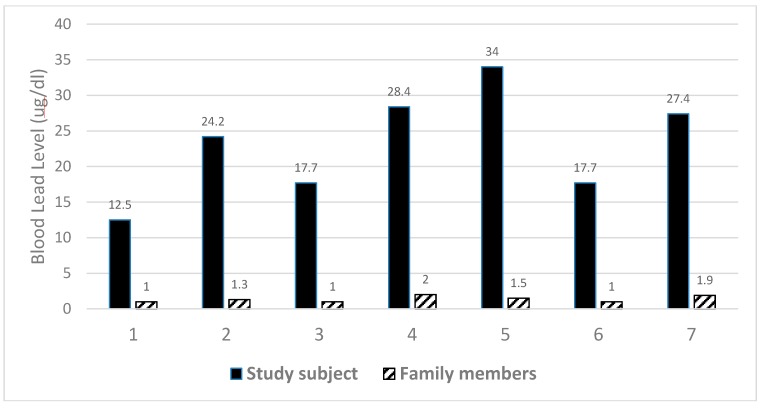
Comparison of current (May 2014) blood lead levels in participants and their family members.

A comparison of seven of the participants with their family members (none of the other family members used Ayurvedic medication) showed significant differences between the two groups with regard to current blood lead concentrations (see Figure 2) (blood samples could not be obtained from the family members of one of the study subjects). The relatively low blood lead concentrations in family members point to a source of lead unrelated to the living environment.

**Table 1:** Profile and outbreak summary by subject.

Study code	Sex	Year of birth	Occupation	Ingestion of Ayurvedic medication	Highest blood lead level	Current blood lead level	Chelation treatment	% body weight loss during acute phase	Symptoms during acute phase	Current health status
1	F	1996	School student	4 capsules/day for 3 months	116	12.5	DMSA >13 courses over two years	12.7	Tiredness, nausea, anemia, abdominal cramps, shortness of breath, severe itching	Ankylosing spondylitis, polycystic ovaries, back, knee and hip aches
2	M	1994	School student	4 capsules/day for 3 months	34	24.2	2 courses (DMSA)	8.6	Nausea, tiredness, emotional instability	Tiredness, headaches, nausea, mood swings, occasional vomiting
3	F	1998	School student	4 capsules/day for 1 month	86	17.7	2 courses (DMSA)	5.5	none	Headaches, stomach pains
4	F	1997	School student	3 capsules/day for 6 months	72	28.4	2 courses (DMSA)	20.0	Abdominal cramps, headaches, soreness over entire body	Knee and back aches
5	M	1997	School student	4 capsules/day for 2 years	66	34.0	2 courses (DMSA)	26.8	Leg pains, loss of appetite, tiredness, difficulty concentrating, irritability, weakness & pain in joints & muscles, clumsiness	Feels “fine”
6	F	1998	School student	4 capsules/day for 18 months	92	17.7	3 courses (one course of Penicillamine, followed by two courses of DMSA)	5.5	Abdominal pain, nausea, anemia, tiredness, lethargy, drowsiness, extreme shortness of breath, vomiting, difficulty concentrating, impaired memory, irritability, weakness & pain in joints & muscles, severe itching, clumsiness	constipation
7	M	1994	School student	4 capsules/day for 4 months	76	27.4	4 courses (DMSA)	4.2	Abdominal pain, tiredness, headaches, difficulty concentrating	Feels “good” but occasional tiredness and abdominal pains
8	F	1986	Non-lead occupation	3 capsules/day for 3 weeks	42	10.4	1 course (DMSA)	10.2	Backache that worsened over time, severe nausea and abdominal cramps, loss of appetite, weakness, confined to bed	Well

### 3.5. Health Status

Prior to the incident the majority of participants described their health as “good” or “excellent.” All reported that they enjoyed active lifestyles and participated in vigorous physical activity, including sports such as rugby, horse riding, marathon running, squash, hockey, and dancing. During the acute phase, subjects experienced a wide range of ill health symptoms and conditions (see Table 1). All the subjects experienced sudden, in some instances severe, weight loss during the acute phase, with body weight reductions ranging from 4% to 34% of their pre-incident body weight. In the current study, ill health symptoms ranged from feeling well without any symptoms to severe symptoms and ill health conditions perceived to be associated with the lead poisoning incident (see Table 1).

### 3.6. Treatment

The study subjects were treated by multiple medical practitioners, who in some respects advised varied treatment regimes, which, to some degree, caused anxiety amongst parents. Chelation treatments were also not readily available in South Africa, leading some parents to seek advice, and import drugs, from the USA. As a result, the cost of treatment for the affected individuals ranged from around R250.00 (USD25) for a locally procured course of DMSA, to around R28 000.00 (USD2800) for imported DMSA. One subject (Case 6 in Table 1) was initially prescribed Penicillamine until DMSA became available. Further details on treatment, by case, are provided in Table 1. In one instance medical insurers declined to pay for the prescribed chelation therapy, leading to the cessation of treatment for the affected individual. The number of courses of chelation therapy administered to a single person ranged from one to more than 13 over a period of two years (see Table 1).

### 3.7. Lead Content of Implicated Ayurvedic Medicine

Laboratory analyses showed that the implicated Ayurvedic medication had an average lead content of 71,208 µg per capsule. At the maximum recommended dose, participants would have ingested around 284,832 µg of lead per day, which is more than 14,241 times higher than the recommended maximum daily intake of 20 µg lead from herbal products in Canada [10].

This study highlights the potential public health risk of using Ayurvedic medicines. The current incident involves the ingestion, mainly by adolescents, of an Ayurvedic medicine for skin problems over a period of up to 18 months without notable evidence of ill health effects. However, within 3 to 4 weeks of commencing the ingestion of a different batch, consumers began experiencing a range of symptoms, such as headaches, abdominal pain, nausea, severe itching, weight loss, and fatigue, which worsened over time. Weaknesses have been revealed in the South African public health system with regard to the time until diagnosis and treatment of lead poisoning. For example, a period of around six months passed between the stage at which participants first started exhibiting symptoms of lead poisoning and seeking medical attention, and the lead poisoning diagnosis in August 2012. During this period several participants continued taking the contaminated Ayurvedic medication. The delayed diagnosis may have been due to a lack of awareness in medical personnel of the usually non-specific signs and symptoms of lead poisoning. Since some study subjects reported their use of Ayurvedic medicines to the wide range of doctors and medical specialists they consulted, a failure to realize the potential role of Ayurvedic medicines in lead poisoning [4] may also have been a contributing factor. The delayed diagnosis resulted in avoidable harm to the study participants, their subjection to a wide range of unnecessary, sometimes invasive, diagnostic tests, and considerable anxiety and expense for their parents. Expertise regarding the treatment of lead poisoning was lacking, and chelation agents were not readily available in South Africa, leading some parents to consult with doctors and also bear the cost of importing chelating agents from the USA.

Despite the absence of a blood lead surveillance program in South Africa, a considerable amount of research has been published to show that certain groups of people in the country are at high risk of lead exposure [9,11,12,13,14,15]. Nevertheless, there appears to be a low level of awareness in the general public, as well as in the medical fraternity, of the signs and symptoms of lead poisoning in general, and the role of Ayurvedic medicines specifically, in lead poisoning. To our knowledge, this is the first published account of an outbreak of lead poisoning from Ayurvedic medicines in an African setting. In this regard, especially in the light of the global upsurge in the use of Ayurvedic medicines [16], there is a pressing need for training and awareness programs regarding the potential health risks of Ayurvedic medicines. Such training and awareness programs should emphasize the potential for lead poisoning from both the intentional use of toxic metals, for example in the *rasa shastra* process, and, as appears to have occurred in the current incident, contamination of a single batch of an Ayurvedic medication (the lead content of capsules from a different production batch was below international guideline levels).

There is a need in South Africa to assess the extent of use of Ayurvedic and other complementary medicines, and the associated risk of lead poisoning. South Africa’s population includes around 1.2 million people (2.5%) of Indian heritage, amongst whom the use of Ayurvedic medicines may be particularly prevalent, and for whom lead hazard awareness campaigns may be particularly important. Concerns have similarly been raised over the toxic metal content of traditional Chinese [17] and African herbal medicines [18]; the latter, in particular, are widely used in South Africa. In these and other communities there is concern that cases of lead poisoning may remain undiagnosed and untreated as a result of limited consciousness within the health sector of the association between Ayurvedic and other traditional herbal medicines, and lead poisoning.

Subsequent to this incident, the South African government, in November 2013, promulgated legislation subjecting complementary medicines to the same regulatory oversight applied to conventional medicines. However, there is resistance within the complementary medicine sector to the new legislation, and limited capacity within the Health Department for enforcement. The legislation is also likely to have limited impact on those who personally or directly acquire Ayurvedic medicines [8], for example through Internet-based purchases or during visits to countries of production.

## 4. Conclusions

The lessons learned through the investigation of this outbreak of lead poisoning from an Ayurvedic medication are of relevance in South Africa and globally, as public health practitioners around the world face a potential increase in cases of lead poisoning from increasing use of Ayurvedic medicines. Given the range of additional sources of lead exposure, the risks of lead poisoning from Ayurvedic medicines in South Africa are best addressed through a comprehensive national lead poisoning prevention strategy and program of action, which is currently absent in the country.
